# Talus morphology differs between flatfeet and controls, but its variety has no influence on extent of surgical deformity correction

**DOI:** 10.1007/s00402-021-03925-w

**Published:** 2021-05-10

**Authors:** Andreas Flury, Julian Hasler, Silvan Beeler, Florian B. Imhoff, Stephan H. Wirth, Arndt Viehöfer

**Affiliations:** Orthopaedic Department, Balgrist University Hospital, University of Zurich, Forchstrasse 340, 8008 Zurich, Switzerland

**Keywords:** Flatfoot, Deformity, Pes planus, Subtalar joint, Valgus

## Abstract

**Background:**

Progressive collapsing foot deformity (PCFD) is a complex 3-dimensional (3-D) deformity with varying degrees of hindfoot valgus, forefoot abduction, and midfoot varus. The first aim of this study was to perform a 3-D analysis of the talus morphology between symptomatic PCFD patients that underwent operative flatfoot correction and controls. The second aim was to investigate if there is an impact of individual talus morphology on the success of operative flatfoot correction.

**Methods:**

We reviewed all patients that underwent lateral calcaneal lengthening for correction of PCFD between 2008 and 2018 at our clinic. Radiographic flatfoot parameters on preoperative and postoperative radiographs were assessed. Additionally, 3-D surface models of the tali were generated using computed tomography (CT) data. The talus morphology of 44 flatfeet was compared to 3-D models of 50 controls without foot or ankle pain of any kind.

**Results:**

Groups were comparable regarding demographics. Talus morphology differed significantly between PCFD and controls in multiple aspects. There was a 2.6° increased plantar flexion (22.3° versus 26°; *p* = 0.02) and medial deviation (31.7° and 33.5°; *p* = 0.04) of the talar head in relation to the body in PCFD patients compared to controls. Moreover, PCFD were characterized by an increased valgus (difference of 4.6°; *p* = 0.01) alignment of the subtalar joint. Satisfactory correction was achieved in all cases, with an improvement of the talometatarsal-angle and the talonavicular uncoverage angle of 5.6° ± 9.7 (*p* = 0.02) and 9.9° ± 16.3 (*p* = 0.001), respectively. No statistically significant correlation was found between talus morphology and the correction achieved or loss of correction one year postoperatively.

**Conclusion:**

The different morphological features mentioned above might be contributing or risk factors for progression to PCFD. However, despite the variety of talar morphology, which is different compared to controls, the surgical outcome of calcaneal lengthening osteotomy was not affected.

**Level of evidence:**

III.

## Introduction

The pathophysiology of the development of adult-acquired flatfoot deformity (AAFD), or more recently termed progressive collapsing flatfoot deformity (PCFD), is not yet fully understood [1]. Although dysfunction of the tibial posterior tendon is widely accepted as a common cause of PCFD, other entities have been identified [1]. These include traumatic [2] or degenerative [3] damage of passive ligamentous structures of the midfoot and hindfoot (e.g. spring ligament), which are key factors of the maintenance of the foot physiology [4–6]. In addition to a decreased arch, there may be valgus angulation of the hindfoot. However, it has been questioned if damage to the above-mentioned structures are really the cause of the valgus deformity [7]. Moreover, talus neck deformity has been identified as the primary cause of the deformity in clubfeet [8]. However, only a few studies investigated changes of the talar morphology [9, 10] or the subtalar joint [7, 11] in PCFD. It is therefore likely that bony morphology might predispose to medial arch collapse by encouraging a plantar shift of the talus [12] as well as to valgus deformity of the hindfoot [7]. Furthermore, no previous study addressed differences in talar morphology and its impact on operative flatfoot correction, especially since the bony correction is done by a reduction movement of the talus in lateral calcaneal lengthening procedures. Overall, the aim to investigate anatomical variants associated with specific foot and ankle pathologies is new in the literature. Understanding morphologic variants might help surgeons on developing more precise forms of corrections in the future.

The purpose of this study was to perform a detailed and comprehensible 3-D analysis of the talus morphology between symptomatic PCFD patients that underwent operative flatfoot correction and controls. We hypothesized that (1) anatomical/morphological differences of the talus may be detected and (2) that its morphology affects the outcome of surgical PCFD correction.

## Materials and methods

The local ethical committee approved this study (Zurich Cantonal Ethics Commission, 2020-01361) and all patients (controls included) gave their informed consent for the use of their data for research purposes. All methods were performed in accordance with relevant guidelines and regulations.

### Design

This study had two aims: (1) to compare the talus morphology between symptomatic flatfeet and controls, and (2) to investigate if morphological differences of the talus affects the deformity correction after lateral calcaneal lengthening procedures. For (1), 3-D talus surface models of PCFD patients and controls were generated and compared. For (2), preoperative and postoperative radiographic parameters of symptomatic flatfeet were collected and correlated to the 3-D talus morphology.

### Study population

Data was collected of patients that were operatively treated at out clinic from January 2008 to July 2018 for PCFD (*n* = 121). Only symptomatic stage II flexible AAFD/PCFD treated with lateral calcaneal lengthening were included in the study. Patients with ankle osteoarthritis were excluded [13, 14]. Other exclusion criteria were additional bony procedures on the hindfoot [i.e. subtalar arthrodesis (*n* = 37) or medial sliding calcaneus osteotomy (*n* = 9)] or the medial column (Cotton osteotomy, arthrodesis of the 1st tarso-metatarsal joint [15, 16] or the navicular-cuneiform joint [17, 18] (*n*, in total = 18)), lack of preoperative MRI or CT of the hindfoot (*n* = 10), and lack of pre- and postoperative complete conventional radiographs (weight-bearing lateral and dorsoplantar views) (*n* = 2) with a minimum follow-up of 3 months.

Finally, 44 caucasian patients with symptomatic stage II pes planus were included (30 females, 14 males). At the time of surgery, the average age was 43.5 ± 11.1 years and the average BMI was 27.6 ± 6.2 kg/m^2^ (Table 1). Mean follow-up was 14.8 ± 11.2 months, of which 33 patients (75%) had a follow-up of 1 year or more (mean 18.8 ± 10.5 months).

**Table 1:** Descriptive statistics of the demographical data and main parameters

	Controls	Symptomatic progressive collapsing foot deformity				Correction achieved	
Number of included cases	*n* = 50	*n* = 44		*n* = 44	*n* = 33		
		Preoperative	*p*-value	3 months Postoperative	1 year Postoperative		*p*-value
Age (years)							
Mean	43.5	46.9	0.27				
SD	11.1	18.1					
Body mass index (kg/m^2^)							
Mean	27.6	28.1	0.68				
SD	6.2	5.6					
Talus neck Torsion (°)							
Mean	23.4	26	0.02*				
SD	5.5	4.9					
Talus neck axis, transversal plane (°)							
Mean	4.8	6.5	0.04*				
SD	6.5	4.2					
Talus neck axis, sagittal plane (°)							
Mean	31.7	33.5	0.04*				
SD	4.8	3.5					
Subtalar joint axis, frontal plane (°)							
Mean	17.9	22.5	0.01*				
SD	9.5	8					
Subtalar joint axis, transversal plane (°)							
Mean	54	52.8	0.48				
SD	8.4	7.3					
Meary’s angle (°)							
Mean		10.9		5.2	4.9	5.6	0.002*
SD		7.9		7.2	6.2	9.7	
Calcaneal pitch (°)							
Mean		17.1		20.5	18.5	1.2	0.31
SD		4.3		4.9	4.4	6.6	
Kite angle (°)							
Mean		47.5		46.5	45.4	2.2	0.23
SD		6.7		6.9	7	10.2	
Talonavicular uncoverage angle (°)							
Mean		22.4		8.9	11.6	9.9	0.001*
SD		13.7		12.6	11.2	16.3	

Continuous are shown as mean ± standard deviation (SD)

Bold values marked with an asterisk are statistically significant

The control group consisted of 50 lower legs of 48 patients (36 males, 14 females), which were assigned out of the department’s database to the study cohort. The controls obtained the CT scan of one or both legs for preoperative planning of surgical procedures around the knee (femoral or tibial mechanical leg axis realignment), using patient-specific instruments. Medical records were reviewed and patients with prior osseous surgery, posttraumatic leg deformity, or foot or ankle pain in any kind, were excluded. The exclusion criteria did not necessarily omit patients with asymptomatic pes planus. However, previous research reported morphologic differences between neutrally aligned feet and symptomatic pes planus, but not when compared to asymptomatic pes planus [9]. As a lot of people are born with flatfeet and are never symptomatic [1], it can be hypothesized that talus morphology in symptomatic pes planus might be a risk factor for progressive deformity and development of pain. Therefore, a diverse cohort of asymptomatic patients (regarding foot and ankle pathology) was considered a suitable control group. Average age and BMI for controls were 46.9 ± 18.1 and 28.1 ± 5.6 (Table 1).

### Surgical technique

According to Hintermann [19], lengthening of the lateral column was done by creating an calcaneal osteotomy from lateral to medial, between the posterior and the medial facet. The medial longitudinal arch was restored by the widening of the Caspar spreader (Hintermann Retractor). The gap was filled with a tailored allograft bone wedge and fixed with one 3.5-mm-cortical screw [19]. Due to tendinopathy, debridement of the tibialis posterior tendon was performed in 5 cases (11.4%). Additional flexor digitorum longus (FDL) tendon transfer was necessary in 25 cases (56.8%) because of severe tibialis posterior tendon degeneration or tear [20].

### Radiographic assessment

Preoperative and postoperative radiographic parameters of the PCFD group were assessed by two senior orthopaedic residents using conventional weight-bearing lateral and dorsoplantar foot radiographs, which included the talometatarsal-angle (Meary’s angle), calcaneal inclination-angle (calcaneal pitch), talocalcaneal-angle (Kite angle), and talonavicular uncoverage-angle [21, 22]. The ICC between the readers were 0.955, 0.987, 0.887, and 0.948 for the talometatarsal-angle, talocalcaneal-angle, calcaneal inclination-angle, and talonavicular uncoverage angle, respectively.

### Three-dimensional (3-D) assessment

CT scans of all PCFD patients and controls were segmented using the global thresholding and region growing functionality of a standard segmentation software (Mimics Medical, Materialise NV, Leuven, Belgium) to generate 3D bone models. Afterwards, the models were imported into the preoperative planning software CASPA (Computer Assisted Surgery Planning Application, Balgrist CARD AG) [23]. All CT scans were performed using a 64-detector row CT scanner (SOMATOM Definition AS Siemens Healthcare, Erlangen, Germany). Slice thickness was 1.0 mm with an inplane resolution of 0.4 × 0.4 mm. Measurement accuracy was 0.01 mm and 0.01° per pixel.

### 3-D measurement of the talus

**Fig. 1:**
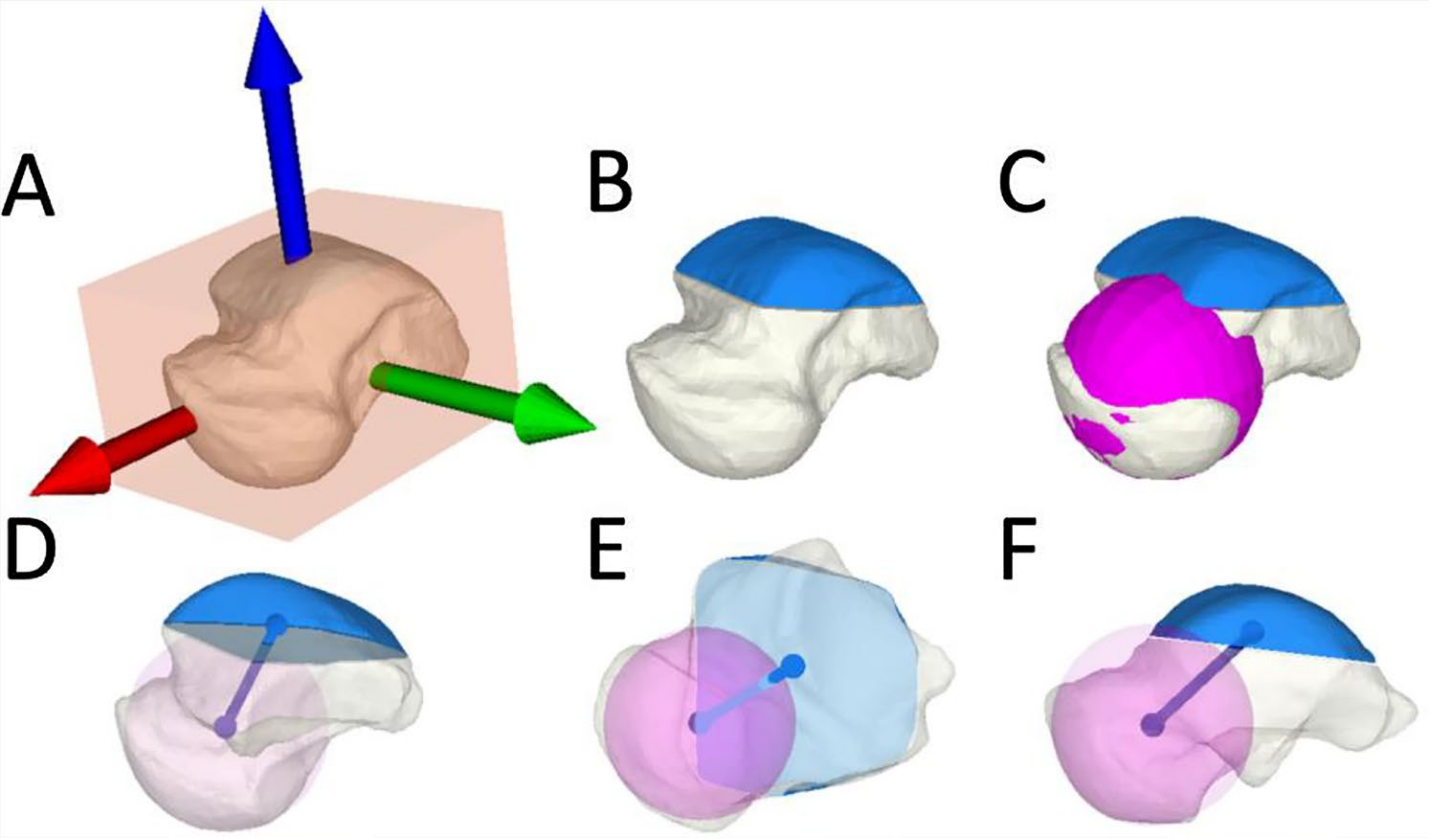
3-dimensional assessement of talus morphology. **a** The talus coordinate system based on the geometric principal axes of the talus. **b** The centre of mass of the superior articular facet of the talus dome was defined (blue mass). **c** A sphere best fitting the talonavicular articular surface was created. **d** The talus neck axis (blue line) connected the centre of mass of the talus dome and the created sphere best fitting the talus head. **e** Superior view and **f** medial view

An oriented bounding box of the talus was generated based on the principal component analysis. Then, a local coordinate system of the talus based on the geometric principal axes was generated (Fig. 1a) [24, 25]. The origin of the talus coordinate system was located in the centroid of the talus, which was again defined by the center of mass. The *X*-axis was pointing anteriorly, the *Y*-axis laterally and the *Z*-axis proximally.

The centre of mass of the superior articular surface of the talus dome was determined, which was defined as the area between the transition from convexity and concavity from talus dome to talus neck anteriorly and to the processus posterior tali posteriorly (Fig. 1b) [26]. The mediolateral borders were defined by the shoulders of the talus dome. A sphere was created and manually adjusted to best fit the talonavicular articular surface (talus head) by the first author (Fig. 1c) [25, 27]. The talus neck axis was defined as the axis between the centre of mass of the superior articular surface of the talus and the centre of the talus head (Fig. 1d–f). Next, a cylinder best fitting the talus dome [26] was created of which the axis defined the axis of the talus dome. Talus neck torsion was defined as the angle of 90° minus the projected angle of the talus neck axis and the talus dome axis, both projected onto the transversal plane of the talus coordinate system. Moreover, the talus neck axis was decomposed in relation to standard axes of the talus coordinate system, according to previous studies [25]. The angle between the projected axis of the talus neck axis and the *X*-axis of the talus coordinate system was projected onto the sagittal and the transversal plane.

The subtalar joint axis was defined as the axis of a cylinder, which best fitted the posterior calcaneal facet of the talus. Subtalar joint axis was decomposed in relation to standard axes of the talus coordinate system as well, using the *Y*-axis. Subtalar joint orientation was assessed as the projected angle between those two axes on the transversal and frontal planes, respectively.

Interobserver ICC and intraobserver ICC for 3-D measurement of the talus neck torsion was 0.886 and 0.956, respectively. For measurement of the subtalar joint orientation in the frontal plane, interobserver and intraobserver repeatability was 0.955 and 0.979, respectively.

### Statistical analysis

All relevant data were entered in a spread-sheet program and statistically analysed with SPSS software version 23.0 (IBM-SPAA, New York, USA). Descriptive and continuous variables were calculated as means ± standard deviation (SD), and range, when appropriate. Mean values are given as the average of both raters. Normal distribution was confirmed with the Kolmogorov–Smirnoff test. Group comparison (flatfeet vs. controls and female vs. male) of preoperative morphologic factors was performed with an unpaired t test. Pearson correlation coefficient was used for continuous variables searching for a correlation between (1) conventional radiographic flatfeet assessment [22] and talus morphology (preoperative 2-D to 3-D parameters), (2) talus morphology and correction achieved (delta between 2-D preoperative and postoperative (three months and one year) parameters correlated to 3-D parameters), (3) talus morphology and loss of correction (delta between 2-D parameters three months and one year postoperatively correlated to 3-D parameters). Rater reliability of all measurements were analysed with intraclass correlation coefficients (ICC) and a two-way model assuming a single measurement and absolute agreement.

## Results

Preoperative values of radiographic parameters are shown in Table 1. Radiographic ples planus deformity showed only a weak correlation to the talus geometry (Table 2). There was a weak but significant correlation of talus body to head relationship and calcaneal pitch (*r* = 0.332, *p* = 0.028), inferior alignment of the talus neck and dorso-plantar talocalcaneal angulation (*r* = 0.367, *p* = 0.014), and frontal subtalar joint orientation and talonavicular uncoverage (*r* = 0.328, *p* = 0.030).

**Table 2:** Correlation analysis (Pearson) of talus morphology and preoperative severity of pes planovalgus deformity

	Meary’s angle	Calcaneal pitch	Kite angle	Talonavicular uncoverage angle
Talus neck torsion	*r* = − 0.039	***r* = 0.332**	*r* = 0.183	*r* = − 0.008
	*p* = 0.800	*p* = 0.028*	*p* = 0.235	*p* = 0.962
Talus neck axis, transversal plane	*r* = 0.000	*r* = -0.057	*r* = − 0.054	*r* = -0.084
	*p* = 1.000	*p* = 0.712	*p* = 0.727	*p* = 0.587
Talus neck axis, sagittal plane	*r* = 0.146	*r* = 0.206	***r* = 0.367**	*r* = 0.117
	*p* = 0.345	*p* = 0.179	*p* = 0.014*	*p* = 0.448
Subtalar joint axis, frontal plane	*r* = 0.049	*r* = 0.079	*r* = 0.200	*r* = 0.328*
	*p* = 0.752	*p* = 0.612	*p* = 0.193	***p* = 0.030**
Subtalar joint axis, transversal plane	*r* = 0.062	*r* = 0.122	*r* = 0.136	*r* = 0.111
	*p* = 0.688	*p* = 0.430	*p* = 0.378	*p* = 0.474

Bold values marked with an asterisk are statistically significant

**Fig. 2:**
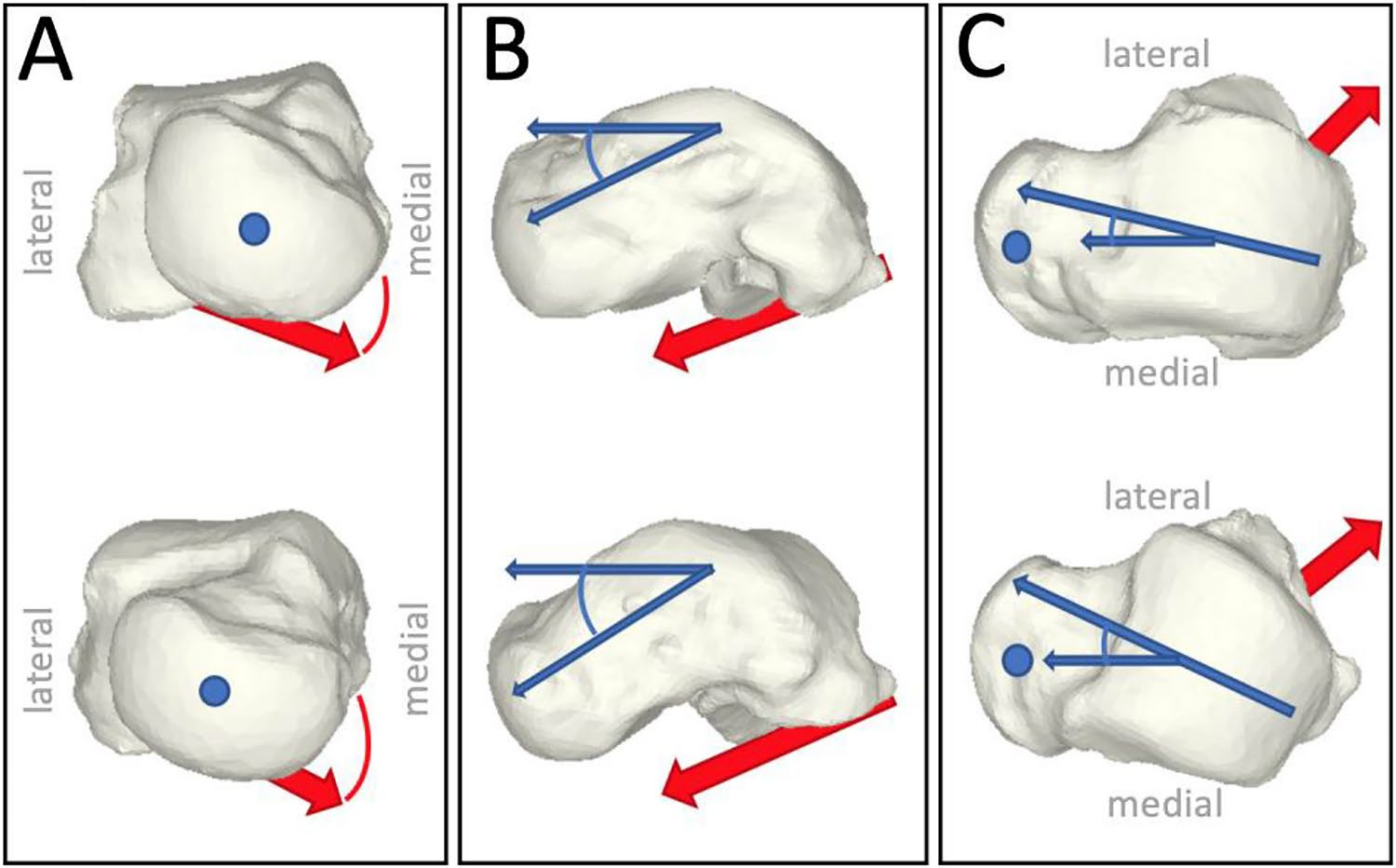
Representative tali of controls (upper row) and symptomatic pes planus (lower row). **a** Aligned according to their coordinate system, which based on their respective geometric principal axes, the subtalar joint orientation (red arrow) showed an increased valgus alignment in frontal view. **b** The talar head to body angular relationship (blue arrows) was significantly increased in cranio-caudal, and **c** medio-lateral direction in symptomatic pes planus versus controls. The blue dot represents the center of the talonavicular articular surface in medio-lateral and cranio-caudal direction

The talar head to body angular relationship differed between PCFD patients and controls in medio-lateral and cranio-caudal direction. There was a 2.6° increased plantar flexion of the talar head in relation to the body in symptomatic pes planus compared to controls (*p* = 0.02) (Table 1; Fig. 2b). Additionally, the talus neck of PCFD deviated a mean of 1.8° medially compared to controls (*p* = 0.04) (Fig. 2c). Moreover, symptomatic flatfeet were characterized by an increased valgus (4.6°) alignment of the subtalar joint, expressed by the orientation of the posterior calcaneal articular facet of the talus (*p* = 0.01) (Fig. 2a). There were no differences in anatomical parameters between male and female subjects in both groups (all n.s.; data not shown).

No influence of talus morphology was found on the correction achieved when comparing PCFD preoperatively and three months or one year postoperatively (data not shown, but all n.s.). In addition, there was no correlation (n.s.) of talus morphology and loss of correction (radiographic PCFD parameters three months postoperatively versus one year postoperatively) (Table 3).

**Table 3:** Correlation analysis (Pearson) of loss of correction (delta 3 months posteropatively and 1 year postoperatively) and talus morphology (*n* = 33)

	Δ Meary’s angle	Δ Calcaneal pitch	Δ Kite angle	Δ Talonavicular uncoverage angle
Talus neck torsion	*r* = 0.062	*r* = 0.096	*r* = 0.071	*r* = − 0.076
	*p* = 0.731	*p* = 0.95	*p* = 0.696	*p* = 0.674
Talus neck axis, transversal plane	*r* = − 0.168	*r* = 0.017	*r* = 0.008	*r* = 0.133
	*p* = 0.349	*p* = 0.925	*p* = 0.963	*p* = 0.459
Talus neck axis, sagittal plane	*r* = 0.163	*r* = 0.095	*r* = 0.104	*r* = 0.162
	*p* = 0.366	*p* = 0.600	*p* = 0.566	*p* = 0.367
Subtalar joint axis, frontal plane	*r* = 0.005	*r* = 0.257	*r* = − 0.104	*r* = − 0.010
	*p* = 0.978	*p* = 0.148	*p* = 0.564	*p* = 0.954
Subtalar joint axis, transversal plane	*r* = − 0.090	*r* = 0.134	*r* = - 0.025	*r* = − 0.203
	*p* = 0.618	*p* = 0.459	*p* = 0.888	*p* = 0.258

*Statistically significant

## Discussion

When flatfoot is acquired during adulthood, the shape of the foot changes. Accordingly, the condition was recently renamed to progressive collapsing foot deformity (PCFD), a complex 3-D deformity with varying degrees of hindfoot valgus, forefoot abduction, and midfoot varus [1]. Evidence exists that anatomical configuration and morphology of the talar bone might predispose to medial arch collaps and be associated with hindfoot malalignment [9]. Louie et al. found a significantly more plantarflexed talar head to body relationship in symptomatic pes planus [9]. In addition, a valgus subtalar joint axis might likely be another contributing or perhaps even primary risk factor for the progression of valgus angulation of the hindfoot [7]. This led to the hypothesis, that further anatomical/morphological characteristics of the talar bone might exist in PCFD, which have been insufficiently investigated so far [27].

In the current detailed and comprehensible 3-D analysis, an overall significant different morphology of the talus between symptomatic flatfeet and controls was found. In addition to Louie et al. [9], the talar head/neck deviated not only caudally but also medially in relation to the talar body. These features might predispose to medial arch collaps by encouraging a plantar and medial shift of the talus, resulting in talonavicular uncoverage and an increased talocalcaneal angle. Moreover, next to the head to body relationship, also valgus orientation of the posterior articular facet of the talocalcaneal joint was significantly increased in symptomatic pes planus, which is in accordance with previous studies [28, 29]. This might contribute to the earlier observed peritperitalar subluxation in PCFD [11].

Nevertheless, the question remains unanswered, if mentioned deformities occurred due to musculotendonous imbalances and subsequent mechanical bone adaption, or if the morphology of the talus leads to PCFD. Concerning the last, one might hypothesize that adult acquired flatfeet become symptomatic with progressive deformity, predisposed through their talus morphology. In contrast, innate pes planus with near-normal tali remain mostly stationary (with regard to deformity) and therefore asymptomatic. However, even though the talar morphology differed between symptomatic flatfeet and controls, there was an only weak correlation to radiographic pes planus assessment (Table 2). This might be due to the different distribution of other risk factors in the present cohort (e.g. overweight). Moreover, only symptomatic flatfeet were radiographically assessed. The small range of values could cause a potential false-negative result. Therefore, adapting the design in further studies might put this into perspective.

Regarding operative management for symptomatic PCFD, it was hypothesized that differences in talar neck and subtalar articular facet morphologies might affect hindfoot correction, especially since the “Chopart joint” and the subtalar joint are anatomically and functionally coupled. By lateral lengthening of the calcaneus, significant radiographic correction of the deformity is achieved, reflected in the improved talometatarsal and talonavicular uncoverage angles [21]. However, no evidence was found that talus morphology might affect surgical correction in our cohort (Table 3). Nevertheless, investigation of morphological variants associated with specific foot and ankle pathologies, and their respective surgical accessibility (corrective potential) is new in the literature, and should be pursued further. This might help surgeons on developing more precise forms of corrections and maybe even prevention of lesions and deformities in the future.

This study should be interpreted in light of its potential limitations. First, this study did not explore talonavicular joint coverage or position of the navicular relative to the talus. One reason was, that only supine CT was used. Weightbearing CT of the foot and ankle is an emerging technology [30]. However, by avoiding cross-articular measurements, no bias was caused by non-weightbearing CT data. Moreover, previous studies dedicated themselves to this topic and were not able to find significant differences regarding the talonavicular coverage between normally aligned feet and symptomatic or asymptomatic pes planus [9, 10]. Therefore, the simplification of using the centre of the talar facet of the talonavicular joint appears reasonable. Next, only the talus was investigated in this study. However, detailed analysis of the cuboid and calcaneus as well as their interaction with the talus should be the focus of future studies. Especially, 3-D weightbearing CT assessment of the hind- and midfoot before and after corrective surgery might be an improved way to assess correlation with the 3-D shape [31]. Furthermore, future work should analyse how these morphological differences are manifest during functional activities. For this, also the correlation to clinical results need to be investigated. Moreover, a bias may be alleged due to the inclusion of only symptomatic stage II flexible AAFD treated with a lateral column lengthening procedure. However, talus morphology was assumed to be relevant in this procedure, since the bony correction is done by a reduction movement of the talus. Therefore, (additional) bony procedures of the medial column that would co-influence radiographic pes planus assessment were excluded. However, further studies should investigate other stages of PCFD as well as other surgical procedures.

## Conclusion

Talus morphology differs between flatfeet and controls, suggesting morphological features to be contributing or risk factors for progression to PCFD. Despite the variety of talar morphology, the surgical outcome of calcaneal lengthening osteotomy in case of symptomatic PCFD was not affected. In the future, assessment of 3-D weightbearing CT data of the hind- and midfoot pre and postoperative are needed.

## Declarations

### Availability of data and materials

The datasets used and analysed during the current study are available from the corresponding author on reasonable request.
